# A Rare Intersection: A Case of Gastric Adenocarcinoma With Yolk Sac Differentiation

**DOI:** 10.7759/cureus.46019

**Published:** 2023-09-26

**Authors:** Sanzida Taslim, Nishat Rimin, Aimen James, Nabila N Anika, Javeria Naz, Abdullah Shehryar, Abdur Rehman

**Affiliations:** 1 Psychiatry, Ross University School of Medicine, Far Rockaway, USA; 2 Internal Medicine, Francis Lewis High School, New York, USA; 3 Dermatology, Rehman Medical Institute, Peshawar, PAK; 4 Surgery, Baylor College of Medicine, Houston, USA; 5 Internal Medicine, Holy Family Red Crescent Medical College and Hospital, Dhaka, BGD; 6 Internal Medicine, Jinnah Sindh Medical University, Karachi, PAK; 7 Internal Medicine, Allama Iqbal Medical College, Lahore, PAK; 8 Surgery, Mayo Hospital, Lahore, PAK

**Keywords:** gastric dysplasia, chemotherapy, adenocarcinoma of stomach, adenocarcinoma, yolk sac tumor

## Abstract

This case report presents a rare instance of a 73-year-old male diagnosed with a yolk sac tumor (YST) coexisting with adenocarcinoma components in the stomach. YSTs are primarily gonadal and seldom occur in extragonadal sites such as the gastrointestinal tract. The patient underwent curative resection followed by chemotherapy, resulting in long-term survival without recurrence. This case contributes to the limited existing literature on gastric YSTs, emphasizing the importance of early diagnosis and effective treatment for this aggressive malignancy. It serves as a valuable addition to our understanding of the pathophysiology, diagnosis, and management of this rare condition.

## Introduction

Yolk sac tumors (YSTs) are primarily known to originate in the gonads, either as standalone tumors or as components of mixed germ-cell tumors. While they are predominantly gonadal, approximately 5% of YSTs manifest in extragonadal sites, including midline structures such as the brain, lungs, upper aerodigestive tract, anterior mediastinum, retroperitoneum, and sacrococcygeal region [1]. Their occurrence in the gastrointestinal tract is exceedingly rare, with only a limited number of cases reported in the stomach in either mixed or pure forms [2]. Remarkably, only a single case has been documented in the esophagus. YSTs often present with a severe clinical course due to widespread metastasis at the time of diagnosis. Previous reports indicate that patients with gastric YSTs often have rapidly fatal outcomes, succumbing to the disease within six weeks, underscoring the aggressive nature of these neoplasms [3].

The objective of this report is to enrich the sparse literature on gastric YSTs with adenocarcinoma components. We present a unique case successfully managed with curative resection and chemotherapy, achieving long-term survival without recurrence. This case underscores the importance of early diagnosis and effective treatment for this rare, aggressive malignancy, serving as a valuable addition to existing research.

## Case presentation

A 73-year-old male presented to the hospital complaining of epigastric pain, anorexia, and melena. The patient had a known history of gastritis and had been taking non-steroidal anti-inflammatory drugs (NSAIDs) for knee joint pain. Upon admission, a comprehensive physical examination was conducted, revealing signs indicative of anemia. Laboratory investigations, including a complete blood count, showed a hemoglobin level of 9.1 g/dL. The constellation of history and clinical findings raised suspicions of gastric carcinoma.

To further evaluate the suspected diagnosis, a gastric endoscopy was performed, revealing an ulcerated and hemorrhagic mass in the antrum of the stomach. A biopsy of the lesion confirmed the histological features of adenocarcinoma. Subsequent imaging studies, including a computed tomography (CT) scan of the chest, abdomen, and pelvis, demonstrated stomach wall thickening and regional lymph node involvement, although no distant metastasis was observed.

Following diagnosis, the patient underwent a total gastrectomy with D2-lymphadenectomy and a subsequent Roux-en-Y gastrojejunostomy. The excised specimen was sent for histopathological analysis, revealing a 50 x 40 mm lesion. The tumor was located 25 mm, 72 mm, 37 mm, and 90 mm away from the proximal, distal, lesser omental, and greater omental margins, respectively. Histopathology confirmed poorly differentiated adenocarcinoma with YST differentiation, extending into the subserosal fat but not invading the visceral peritoneum or nearby structures. Of 16 lymph nodes examined, 13 were involved, and lymphovascular invasion was present. The pathological stage was classified as T3N3aM0 stage III gastric adenocarcinoma with YST differentiation, per the American Joint Committee on Cancer (eighth edition).

**Figure 1:**
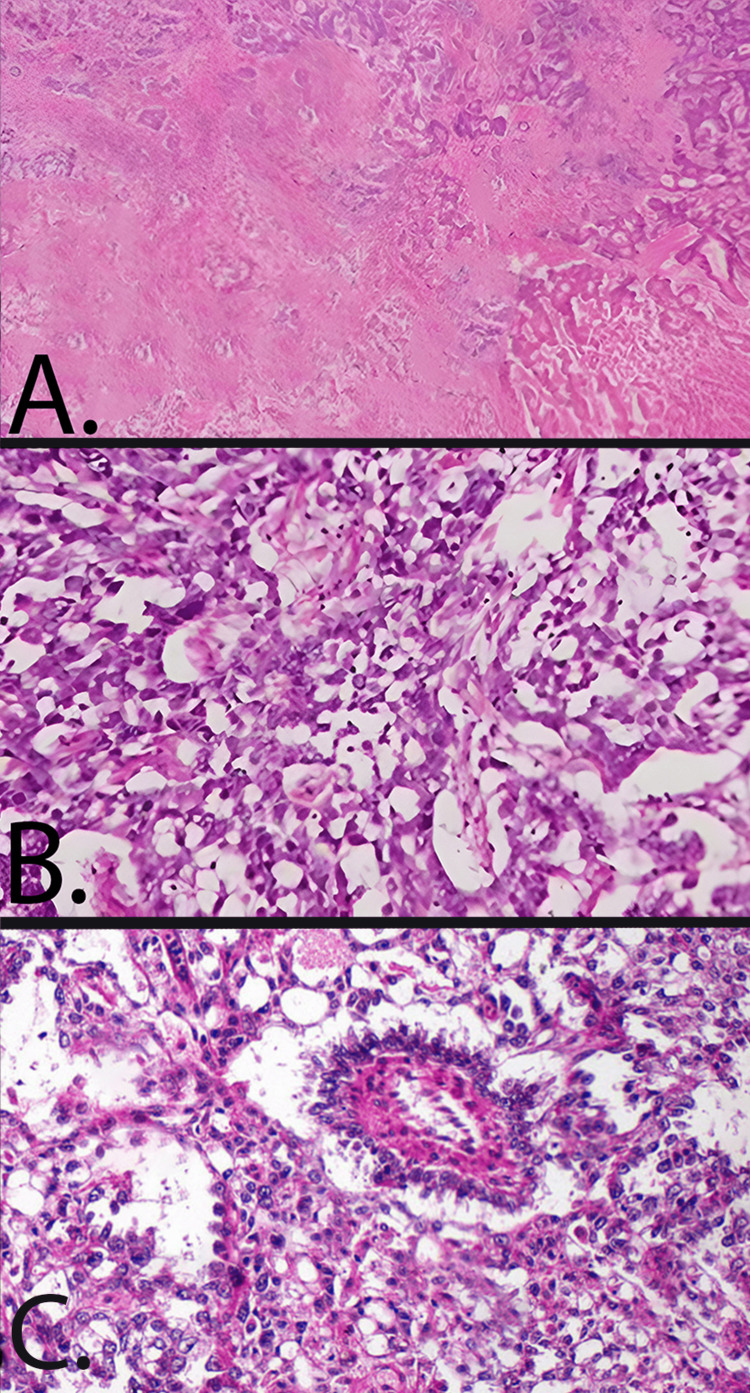
Adenocarcinoma and yolk sac tumor (YST) found during a histological investigation. The YST exhibits a reticular pattern shown in panels A and B. Schiller-Duval bodies are evident in panel C (hematoxylin and eosin staining).

Microscopically, the adenocarcinoma component consisted of poorly differentiated tubular structures. The YST component exhibited a reticular pattern lined by a variable combination of flattened malignant cells, papillary structures, and cuboidal cells. Immunohistochemical staining revealed that the YST component was SALL4-positive and contained glycogen and hyaline globules. Schiller-Duval bodies were sporadically present. Additionally, the YST component tested positive for p53, carcinoembryonic antigen, and alpha-fetoprotein (AFP), while the adenocarcinoma component was only p53-positive. Features obtained on biopsy are elaborated in Figure 1.

Following surgical intervention, the patient was referred to the oncology department for further management. After a thorough review of the patient’s history, investigations, and surgical findings, adjuvant chemotherapy was initiated. The regimen consisted of four cycles of carboplatin at a dosage of AUC 3 and paclitaxel at 100 mg/m^2^, both administered bi-weekly (q2 weekly). The patient demonstrated a favorable response to the treatment, showing no signs of disease recurrence on regular CT scan follow-ups for 18 months post-chemotherapy initiation. Given the absence of any features of recurrence on the follow-up CT scans, the same chemotherapeutic regimen was continued, with a strong emphasis on regular monitoring and follow-up.

## Discussion

Choriocarcinoma and YST-like differentiation in gastric cancers are exceedingly rare. While there have been reports of gastric adenocarcinomas concurrently possessing choriocarcinoma and YST components, our case specifically discusses the coexistence of adenocarcinoma and YST differentiation in the stomach, adding to the limited literature on this topic [1]. The pathophysiology of germ-cell tumors originating in non-gonadal organs such as the stomach remains elusive. Hypotheses suggest that these tumors may arise from multipotent neoplastic epithelial cells in the gastric mucosa or from migratory germ cells during embryonic development [2]. Some research posits that the stomach, which originates from the foregut, retains all genetic material in its cells, allowing tumor cells to de-differentiate into a yolk sac phenotype [3].

Men, with a mean age of 65.2 years, are more susceptible to gastric YSTs than women. The clinical symptoms often include epigastric discomfort, hematemesis, anorexia, abdominal fullness, and weight loss, but these are generally non-specific [4]. Earlier studies have reported only two cases of pure YSTs without adenocarcinoma, while most patients presented with malignant germ-cell tumors and adenocarcinoma [5]. The case under discussion also highlights a malignancy comprising both adenocarcinoma and germ-cell tumors.

Elevated serum AFP levels, commonly seen in YSTs, usually decline post-resection but remained stable in our case, suggesting their potential as a predictive marker for recurrence and survival [6]. The generally poor prognosis of gastric adenocarcinomas with YST elements, often metastasizing to lymph nodes, lungs, or liver, further underscores the importance of AFP levels as a diagnostic clue, especially when gonadal tumors and hepatocellular carcinoma are ruled out [7].

The optimal treatment strategy beyond early diagnosis and curative resection remains undetermined. More research and clinical data are needed to establish the most effective treatment for gastric YSTs. To date, only 20 cases have been reported [8]. To our knowledge, the case currently being discussed is the 21st case of its nature. The cases described in the literature have been summarized in Table 1.

**Table 1:** Compilation of cases featuring yolk sac tumors, either isolated or in conjunction with adenocarcinoma, as documented in the literature. YST: yolk sac tumor; AC: adenocarcinoma; CC: choriocarcinoma; HC: hepatocellular carcinoma; LN: lymph node; S: surgery; CT: chemotherapy; NM: mot mentioned; RFA: radiofrequency ablation

Authors (year)	Age (years)/Sex	Histology	Metastasis	Therapy	Prognosis
Garcia et al. 1985 [3]	65/M	YST, CC, AC	Liver	None	Autopsy case
Motoyama et al. 1985 [9]	72/F	YST, AC	LN	S	Alive (3 years)
Zámecník et al. 1993 [5]	88/M	YST	LN, Peritoneum	S	Died (4 weeks)
Suzuki et al. 1999 [10]	56/M	YST, AC	LN	S, CT	Died (6 months)
Puglisi et al. 1999 [11]	61/M	YST, AC	Peritoneum	Palliative S	Died (1 months)
Wang et al. 2000 [8]	36/M	YST, AC	LN	CT	Died (6 months)
Napaki 2004 [12]	38/F	YST, AC	Liver	CT, S	Alive (32 months)
Kanai et al. 2005 [13]	87/M	YST	None	S	Died (7 months)
Singh et al. 2007 [14]	67/M	YST, AC	Liver, LN	S, CT	Died (2 months)
Tahara et al. 2008 [15]	74/M	YST	Liver, lung, LN	None	Died (6 days)
Kim et al. 2009 [16]	61/M	YST	None	S	Alive (3 months)
Magni et al. 2010 [17]	62/M	YST	LN	S, CT	Died (1 year)
Satake et al. 2011 [1]	74/M	YST, CC, AC	Liver, LN	S, RFA, CT	Alive (8 months)
Bihari et al. 2013 [7]	50/M	YST, AC	Liver	None	NM
Yalaza et al. 2017 [18]	68/F	YST, AC	LN	S, CT	Died (8 months)
Lakshmanan et al. 2017 [19]	75/M	YST, AC, HC	None	S	Alive (30 months)
Qureshi et al. 2018 [20]	52/M	YST, AC	LN	S, CT	Alive (16 months)
Mandelia et al. 2018 [21]	3/M	YST	Liver, peritoneum	CT, S	Alive (5 months)
Ibrahim et al. 2019 [22]	86/F	YST	None	S	NM
Umeda et al. 2021 [23]	77/M	YST, AC	None	S	Alive (7 years)
Present case (2021)	73/M	YST, AC	None	S, CT	Alive (18 months)

One of the limitations of this case report is the absence of endoscopic images of the stomach. While we were unable to procure the endoscopy images, we have included detailed histopathological biopsy pictures, which provide valuable insights into the tumor’s characteristics. These images, combined with the comprehensive clinical, surgical, and histological data presented, offer a thorough understanding of this rare case.

Adjuvant chemotherapy agents such as cisplatin, vinblastine, bleomycin, and etoposide have not proven effective in enhancing long-term survival for gastric YSTs, which are typically chemotherapy-resistant [8]. Current research on targeted therapies is limited. In our case, a multidisciplinary team debated treatment options. Due to the rarity of similar cases, selecting the optimal treatment was challenging. Contrary to some studies indicating poor chemotherapy outcomes, our patient showed a favorable prognosis post-chemotherapy.

## Conclusions

The confluence of adenocarcinoma and YST differentiation in gastric cancers is exceptionally rare, leaving its exact pathogenesis largely uncharted. Generally linked to poor outcomes, early diagnosis via routine screenings could improve prognosis. The ideal treatment approach, combining medical and surgical interventions, remains undetermined.

This case report holds multifaceted value for medical literature. It augments sparse biostatistical data, aiding future research. It also sheds light on the pathophysiology of a complex, poorly understood malignancy and serves as a clinical guide for similar cases. Thus, this report is a pivotal addition to existing studies, aiming to catalyze further research and enhance patient care.
